# Protocol for detecting cancer in blood using targeted DNA methylation

**DOI:** 10.1016/j.xpro.2026.104791

**Published:** 2026-08-20

**Authors:** Zhaoyu Jiang, Yuqing Guo, Yongqu Lu, Kun Qian, Renjie Luo, Xiaomeng Liu, Jie Ren, Wei Fu, Lu Wen, Xin Zhou

**Affiliations:** 1 Department of Gastrointestinal Surgery, Central Hospital Affiliated to Shandong First Medical University, Jinan 250000, China; 2 Department of General Surgery, Cancer Center, Biomedical Pioneering Innovation Center, Third Hospital, Peking University, Beijing 100871, China; 3 Beijing Advanced Innovation Center for Genomics (ICG), Ministry of Education Key Laboratory of Cell Proliferation and Differentiation, School of Life Sciences, Peking University, Beijing 100871, China; 4 Department of Breast and Thyroid Surgery, China-Japan Friendship Hospital, Beijing 100029, China; 5 School of Mathematical Sciences and Center for Statistical Science, Peking University, Beijing 100871, China; 6 Radiation Oncology Key Laboratory of Sichuan Province, Sichuan Cancer Hospital &Institute, Chengdu 610041, China

**Keywords:** Cancer, Health Sciences, Genomics, Sequencing, Molecular Biology, Gene Expression, Biotechnology and bioengineering

## Abstract

Here, we present a protocol for detecting and differentiating colorectal and gastric cancers using plasma cell-free DNA (cfDNA) with targeted methylated CpG tandem amplification and sequencing (tMCTA-seq), a targeted DNA methylation sequencing approach. We describe steps for sample collection, DNA extraction, bisulfite conversion, and targeting 110 methylated sites by coupling locus-specific primers with a universal CGCGCGG primer. We then detail an optional workflow that adapts tMCTA-seq to other cancer types. This approach achieves high sensitivity, below one haploid-genome equivalent, enabling non-invasive cancer detection.

## Highlights

• Steps for CRC/GC detection using targeted methylation sequencing of CGCGCGG sites
• Procedures for bisulfite conversion and linear amplification with UMI tagging
• Guidance on multiplex-nested PCR and library preparation within 8 h
• Instructions for bioinformatics analysis to differentiate CRC from GC in blood

## Abstract (editor highlights)

Publisher’s note: Undertaking any experimental protocol requires adherence to local institutional guidelines for laboratory safety and ethics.

## Graphical abstract

**Figure:**
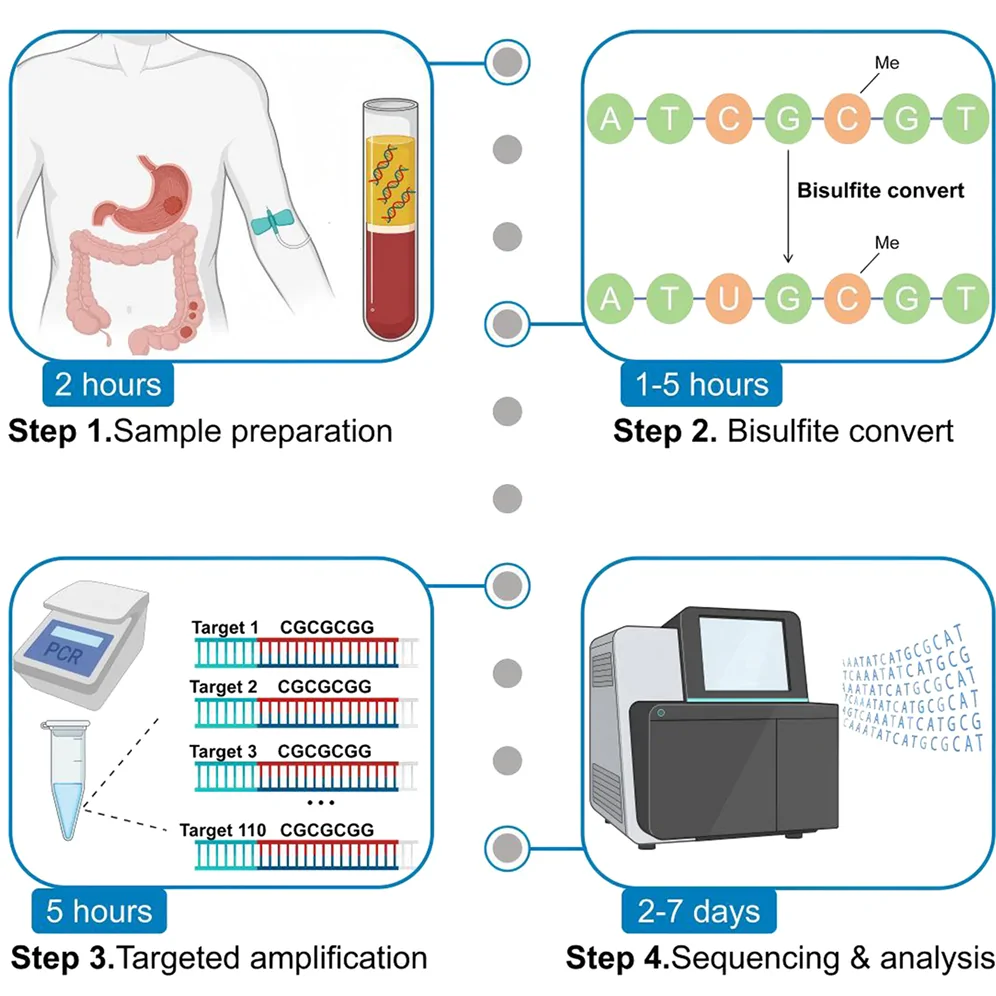

## Before you begin

Colorectal cancer (CRC) and gastric cancer (GC) present significant global health burdens with high mortality rates.^1^ Aberrant DNA methylation is a prevalent phenomenon in human cancers and, owing to its stability, serves as an excellent biomarker for the disease. Liquid biopsy, which detects circulating tumor DNA (ctDNA) in the blood, offers a non-invasive approach for cancer detection. Unlike point mutations, which are often patient-specific, aberrant DNA hypermethylation at specific loci occurs in a high percentage of cancers. This makes DNA methylation-based liquid biopsy particularly useful for early cancer screening, companion diagnostics, and tumor-agnostic minimal residual disease monitoring.^2^^,^^3^^,^^4^ This protocol outlines targeted methylated CpG tandem amplification and sequencing (tMCTA-seq), a cost-effective and highly sensitive targeted DNA methylation assay. It utilizes a single-sided multiplex-nested PCR design to target a panel of 110 methylated sites for the simultaneous detection and differentiation of CRC and GC in blood.

The specific procedures below describe sample preparation for clinical detection using approximate 2 mL of plasma, after bisulfite conversion and three-steps PCR-based targeted amplification, and enable library preparation within 8 h using common next-generation sequencing instruments.

### Innovation

tMCTA-seq is a PCR-based targeted DNA methylation method. In contrast, hybridization capture has been more commonly used to target multiple DNA methylation loci for ctDNA detection. A prominent example is the Galleri test, which captures over 100,000 regions for pan-cancer screening.^5^ However, when targeting a modest number of loci, PCR-based methods can be simpler, faster, and more cost-effective than hybridization capture methods.

tMCTA-seq was developed from MCTA-seq, a genome-scale DNA methylation method that examines approximately 9,000 CpG islands (CGIs) and 20,000 CGCGCGG sites.^6^ Because tMCTA-seq targets the same mCGCGCGG-CpG sites as MCTA-seq, it enables an efficient development strategy in which MCTA-seq is first used for unbiased marker discovery, followed by the establishment of a targeted tMCTA-seq assay. For each mCGCGCGG-CpG marker, a locus-specific primer upstream of the CGCGCGG sequence is designed and tested to build a cost-effective targeted assay. This strategy differs from the typical workflow used to develop standard multiplex PCR-based methylation assays, in which markers are usually selected from methylation array or methylome sequencing data generated from tumor tissues, adjacent normal tissues, and peripheral blood leukocytes, followed by the design of a pair of locus-specific primers for each target. Compared with this standard approach, the MCTA-seq/tMCTA-seq strategy specifically focuses on hypermethylated CGCGCGG loci and offers two distinctive advantages.

First, the MCTA-seq/tMCTA-seq workflow enables clearer evaluation of the clinical performance of candidate loci during marker screening. Second, the single-sided multiplex-nested PCR design of tMCTA-seq simplifies primer design and PCR setup, allows straightforward incorporation of unique molecular identifiers (UMIs) for accurate molecule counting, and is well suited to the short fragment size of ctDNA because it relies on the short CGCGCGG sequence. Together, these features enable robust capture of trace amounts of ctDNA from plasma, with a technical sensitivity of less than one haploid genome equivalent, and support the simultaneous detection and differentiation of CRC and GC.

### Institutional permissions

The Ethics Committee of Peking University Third Hospital approved this study (IRB00006761-2016003). All participants provided written informed consent prior to sample collection. Ensure compliance with your institutional review board or local ethics committee guidelines for human sample collection and usage before implementing this protocol.

### Sample collection and DNA extraction

**Timing: 2 h**

Extract cfDNA from plasma for clinical testing.

Differential centrifugation separates blood into three density-based layers: (1) the upper plasma layer containing circulating cell-free DNA (cfDNA) and proteins; (2) the middle buffy coat, composed of peripheral blood mononuclear cells (PBMC); and (3) the lower red blood cell and granulocytes. For cfDNA analysis, only the plasma layer (upper) should be collected. The buffy coat layer contains a large number of leukocytes and therefore must be carefully avoided during plasma collection; otherwise, their genomic DNA (gDNA) will dilute the ctDNA signal and impair assay sensitivity. The second centrifugation of plasma layer at higher speed further removes residual cellular debris, platelets, and other particles that might interfere with downstream analysis.

1. Isolate plasma and WBCs.
  a. Collect 4 mL of peripheral blood into K2-EDTA tubes.
  b. Centrifuge at 1,350 × *g* for 10 min at 4 °C using a swing-bucket rotor with slow acceleration and deceleration.
  c. Carefully transfer the upper clear-to-yellow plasma layer to a new 2 mL tube, avoiding the buffy coat.
  d. Collect the buffy coat layer (containing PBMCs) into a separate 2 mL tube.
  e. Centrifuge the isolated plasma again at 16,000 × *g* for 10 min at 4 °C to remove residual debris. Transfer the supernatant to a fresh tube.
  f. Store all clarified plasma and buffy coat aliquots at −80 °C until further processing.

**CRITICAL:** If plasma isolation cannot be executed within 6 h of phlebotomy, blood should be collected in specialized cfDNA preservation tubes (e.g., Streck Cell-Free DNA BCT).

**Pause point:** Store plasma and buffy coat aliquots at −80 °C until cfDNA extraction.

2. Extract cfDNA from plasma samples.
  a. Extract cfDNA from 2 mL of plasma using a standard commercial kit (e.g., QIAamp Circulating Nucleic Acid Kit) according to the manufacturer’s instructions.
  b. Elute the cfDNA in 20–30 μL of 10 mM Tris-HCl (pH 8.0) buffer with low EDTA (0.1 mM). Nuclease-Free Water is acceptable.
  c. Quantify the extracted cfDNA using a fluorometric assay (e.g., Qubit dsDNA HS Assay Kit) according to the manufacturer’s protocol.
3. Extract gDNA from WBCs for background controls.
  a. Extract gDNA from the collected buffy coat using a standard commercial kit (e.g., DNeasy Blood & Tissue Kit) according to the manufacturer’s instructions.
  b. Elute the gDNA in 100 μL of Nuclease-Free Water or the kit-provided elution buffer containing low EDTA (0.1 mM).
  c. Quantify the extracted gDNA using the Qubit dsDNA HS Assay Kit.

***Note:*** Most standard cfDNA extraction kits are suitable for this protocol. We validated the VAHTS Free-Circulating DNA Maxi Kit (Vazyme, N903), and also confirmed that the QIAamp MinElute ccfDNA Mini Kit (QIAGEN, 55204) is applicable. Spectrophotometric assays are not recommended for cfDNA quantification due to their limited sensitivity and specificity.

**Pause point:** Store at −80 °C until bisulfite conversion.

### DNA pre-treatment, conversion, and control setup

**Timing: 1–5 h**

Bisulfite treatment converts unmethylated cytosines to uracils, while 5-methylcytosines remain unchanged. This differential conversion allows for methylation detection: unmethylated cytosines become T after PCR, while methylated cytosines remain as C. Sequencing then reveals the methylation status based on C vs. T presence, converting epigenetic signals into detectable DNA sequence differences. Specifically, in tMCTA-seq, bisulfite treatment converts unmethylated CGCGCGG to TGTGTGG, while the methylated CGCGCGG remain unchanged, allowing selective amplification of methylated loci.Perform bisulfite conversion on clinical cfDNA samples in parallel with positive and negative controls. Use WBC gDNA as the negative control, and prepare the positive control by spiking Fully Methylated Genomic DNA (FMG) into WBC gDNA.

4. Perform bisulfite conversion for clinical cfDNA samples.
  a. Add 100 ng of Carrier RNA to the sample prior to conversion if the initial cfDNA input amount is < 6 ng.
  b. Bisulfite-convert the cfDNA samples using the EpiArt Ultrafast DNA Methylation Bisulfite Kit according to the manufacturer’s instructions. The EZ DNA Methylation-Lightning Kit (ZYMO) bisulfite conversion kits has been validated to be applicable.
  c. Elute the converted cfDNA in 22 μL of Nuclease-Free Water.

***Note:*** The time required for bisulfite conversion varies by kit. Recently developed high-concentration bisulfite conversion kits can complete the process in 1 h.^5^ Recently reported enzymatic conversion using a single enzyme could also offer a more convenient alternative.^6^ Library construction can proceed without quantifying the post-conversion single-stranded DNA (ssDNA) yield.

5. Fragment gDNAs for control samples.
  a. Prepare 1 μg of extracted WBC gDNA and 1 μg of FMG (e.g., Universal Methylated Human DNA Standard, Zymo Research, Cat# D5011) into appropriate processing tubes.
  b. Shear the DNA using an acoustic fragmentation instrument (e.g., Covaris focused-ultrasonicator) to a target peak size of 150–200 bp.
  c. Verify the fragment size distribution using a capillary electrophoresis system (e.g., Agilent 5200 Bioanalyzer with High Sensitivity DNA Kit).

***Note:*** The acoustic fragmentation method has been validated in this protocol. Alternative fragmentation methods, such as enzymatic digestion, may also be considered, provided that they do not introduce sequence- or base-composition bias. However, these methods should be independently optimized and validated before use. For the Covaris M220 focused-ultrasonicator, optimal settings are: Peak Incident Power 50 W, Duty Factor 20%, Cycles per Burst 200, Treatment Time 330 s, Temperature 20 °C. For fragment analysis, capillary electrophoresis is recommended because it provides higher size resolution and greater sensitivity for detecting small DNA fragments. If capillary electrophoresis systems are unavailable, high-sensitivity agarose gel electrophoresis is an acceptable alternative, particularly for quality assessment rather than absolute quantification.

6. Perform bisulfite conversion for control samples.
  a. Convert the fragmented FMG and WBC gDNA separately using the same bisulfite conversion kit and protocol as used for the cfDNA samples in step 4.
  b. Elute in an appropriate volume (e.g., 10–15 μL) of Nuclease-Free Water.
  c. Quantify the concentration of the converted ssDNA using the Qubit ssDNA Assay Kit.
7. Prepare the positive and negative control samples.
  a. Spike 30 pg converted FMG into 6 ng of converted WBC gDNA.
  b. The WBC gDNA is used as the negative control.

**CRITICAL:** High-concentration FMG is a potent source of cross-contamination. Strict spatial separation is mandatory. Process all clinical and negative control samples in a dedicated pre-PCR environment prior to handling any FMG aliquots. Decontaminate all work surfaces and reusable equipment with DNA-Off or an equivalent DNA-degrading solution after use; alternatively, wipe with freshly prepared 0.5–1% sodium hypochlorite followed by 70% ethanol. UV decontamination may also be applied when appropriate. Use dedicated lab coats and fresh gloves for FMG handling, and discard all disposable tips and tubes that have contacted FMG.

### Marker discovery and locus-specific primer design

**Timing: Variable**

To empower researchers to adapt this protocol for other cancer types, we outline the pipeline for identifying novel mCGCGCGG-CpG loci and designing corresponding locus-specific primers. This entire procedure builds upon the MCTA-Seq profiling methodology, with the exact workflows, execution code, and statistical filtering parameters being described.^7^^,^^8^^,^^9^

8. Marker selection.
  a. Differential hypermethylation in tumors: Perform genome-wide methylation profiling on paired primary tumor tissues (e.g., CRC or GC) and adjacent normal mucosa to identify cancer-specific hypermethylated mCGCGCGG-CpG loci.
  b. High methylation signals in tumors: Select loci that exhibit high median methylation values in cancer tissues.
  c. Low cfDNA background in normal plasma: Select loci that exhibit low methylation frequencies in cfDNA of healthy individuals.
  d. Short target length: Select loci where the target CpG sites are located within 60 bp downstream of the CGCGCGG motif.
9. Locus-specific primer design.
  a. For each selected locus, design a specific primer for the single-sided multiplex-nested PCR. Calculate the annealing temperature (Tm) using the NEB Tm calculator (https://tmcalculator.neb.com), configuring the reaction for Taq DNA polymerase at a 20 nM primer concentration.
  b. To ensure uniform multiplexing compatibility and optimal amplification efficiency, strictly adhere to the following five design criteria:
    i. GC content: 50%–60%.
    ii. Primer length: 20–28 bp.
    iii. Tm: 60 ± 3 °C.
    iv. Proximity to target: The distance between the 3′ end of the primer and the 5′ end of the CGCGCGG sequence must be < 20 bp to accommodate the highly fragmented nature of cfDNA.
    v. 3′-Terminal specificity: The 3′ end preferably terminates with CGC (corresponding to the genomic sequence immediately upstream of the CGCGCGG motif) while strictly avoiding the sequence GGCGC. Alternatively, the 3′ end may terminate with a single C (corresponding to the 5′ end of the CGCGCGG motif).

***Note:*** After primer design, target primers should be evaluated using positive and negative controls. The positive control consists of 6 ng of bisulfite-converted WBC gDNA spiked with 150 pg of converted FMG (∼50 molecules), whereas the negative control consists of 6 ng of bisulfite-converted WBC gDNA alone. These controls enable assessment of the absolute capture rate and background signal for each locus. Primers showing low capture rates should be redesigned. In our experience, the capture rate can exceed 50% for well-performing primers, and we generally retain primers with a capture rate greater than 10%. Some loci do not yield suitable primers and therefore must be discarded. Evaluating pooled primers improves the efficiency of this process.

## Key resources table

**Table:** 

REAGENT or RESOURCE	SOURCE	IDENTIFIER
**Biological samples**		

Universal Methylated Human DNA Standard (FMG)	Zymo Research	D5011

**Chemicals, peptides, and recombinant proteins**		

dNTP/dUTP Mix (4 mM dUTP, 2 mM each dATP, dCTP, dGTP)	Thermo Fisher Scientific	R0251
Klenow Fragment (3'→5′ exo-)	New England Biolabs (NEB)	M0212
Uracil-DNA Glycosylase (1 U/μL)	Thermo Fisher Scientific	EN0362
Ethanol	HUSHI	10009218
Nuclease-Free Water	Thermo Fisher Scientific	AM9932
AMPure XP DNA Cleanup Beads	Beckman Coulter	A63882

**Critical commercial assays**		

DNeasy Blood & Tissue Kit	Qiagen	69506
VAHTS Free-Circulating DNA Maxi Kit	Vazyme	N903
EpiArt Ultrafast DNA Methylation Bisulfite Kit	Vazyme	EM112
KAPA HiFi HotStart Uracil+ReadyMix Kit	Roche	KK2802
TaKaRa Ex Taq Hot Start Version	Takara	RR006
Qubit ssDNA Assay Kit	Thermo Fisher Scientific	Q10212
Qubit dsDNA Assay Kit	Thermo Fisher Scientific	Q32854
EzyNGS UDI primer for illumina	Sangon Biotech	N608312-0384

**Deposited data**		

Raw sequence data	Jiang et al.^1^	GSA-Human: HRA004986 (BioProject: PRJCA018045)

**Oligonucleotides**		

Primer A (5 μM, equimolar mixture of Primers 1, 2, 3, 4) 1: TTTCTCATTCTTCACAATACACATCTTACTTTCCCT ACACGACGCTCTTCCGAUCUHHHHHHHHCGCH 2: TTTCTCATTCTTCACAATACACATCTTACTTTCC CTACACGACGCTCTTCCGAUCUHHHHHHHCGHCH 3: TTTCTCATTCTTCACAATACACATCTTACTTTCCC TACACGACGCTCTTCCGAUCUHHHHHHCGHHCH 4: TTTCTCATTCTTCACAATACACATCTTACTTTCCCTA CACGACGCTCTTCCGAUCUHHHHHCGHHHCH	Sangon Biotech (HPLC-purified)	N/A
Primer B: ACTTTCCCTACACGACGCTCT	Sangon Biotech (HPLC-purified)	N/A
Primer C: GTGACTGGAGTTCAGACGTGTGCT CTTCCGATCTDDDDCGCGCGG	Sangon Biotech (HPLC-purified)	N/A
Primer D:CAAGCAGAAGACGGCATACGAGAT XGTGACTGGAGTTCAGACGTGTGCT (X represents the 8-bp i7 index sequence)	Sangon Biotech (HPLC-purified)	N/A
Primer E: AATGATACGGCGACCACC GAGATCTACACXACACTCTTTCCCTA CACGACGCTCTTCCGATCT (X represents the 8-bp i5 index sequence)	Sangon Biotech (HPLC-purified)	N/A
110 locus-specific primers, see Table S1	Sangon Biotech (ULTRAPAGE-purified)	N/A

**Software and algorithms**		

Analysis code	This paper	https://github.com/Guo-Yuqing/tMCTA-seq
Cutadapt software (v3.4)	This paper	https://cutadapt.readthedocs.io/en/v3.4/
Fastp (v0.23.0)	This paper	https://github.com/OpenGene/fastp
Bismark (v0.16.3).	This paper	https://github.com/FelixKrueger/Bismark
Samtools (v1.3.1).	This paper	https://github.com/samtools/samtools

**Other**		

BD Vacutainer EDTA Tubes	BD Biosciences	366643
Qubit Flex Fluorometers	Thermo Fisher Scientific	Q33327
M220 Focused-ultrasonicator	Covaris	500295
DNA LoBind Tubes, 1.5 mL	Eppendorf	0030108051
DNA LoBind Tubes, 2.0 mL	Eppendorf	0030108078
Hieffimag Separation Rack	Vazyme	CM101/103
PCR 8-Strip Tubes	Axygen	PCR-0208-C
PCR 8-Strip Flat Caps	Axygen	PCR-2CP-RT-C
Filter Tips (10 μL, 20 μL, 200 μL, 1000 μL)	KIRGEN	KG5031/5532/5233/5333
5200 Fragment Analyzer System	Agilent Technologies	M5310AA
NovaSeq X Sequencing System	Illumina	20084803

## Step-by-step method details

### Linear amplification

**Timing: 1.5 h**

**Figure 1:**
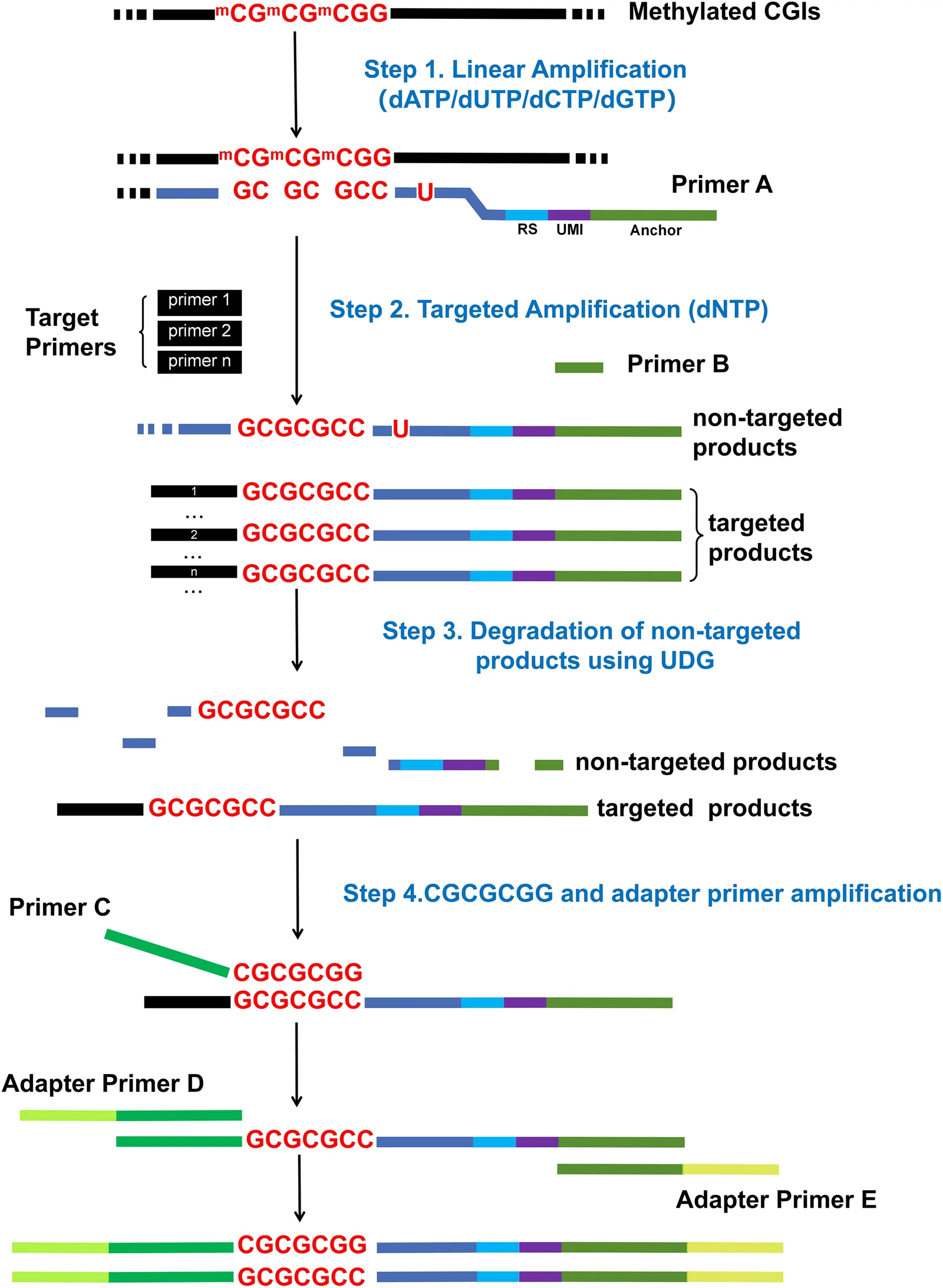
Schematic of tMCTA-seq Following bisulfite conversion, linear amplification using Primer A Mix (featuring a UMI and 3′ semi-random sequence) and a dNTP/dUTP mix preferentially enriches methylated CpG regions while incorporating uracils into amplicons. Next, multiplexed targeted PCR (Target Primers and Primer B) is performed, followed by UDG treatment to selectively degrade dUTP-containing residual primers and off-target products. Finally, terminal PCR using Primer C (targeting the CGCGCGG motif) and indexed adapter primers (Primers D and E) generates the multiplexed sequencing library.

***Note:*** The dNTP/dUTP mix contains dATP, dCTP, dGTP, and dUTP, typically formulated with a higher concentration of dUTP (e.g., 4 mM dUTP *vs.* 2 mM others dNTPs) to ensure efficient uracil incorporation. The Primer A Mix is an equimolar blend of four primers (see key resources table). Its “H” sequence acts as a Unique Molecule Identifier (UMI) (H = A/T/C). Together with the 3′ semi-random sequence, this 12-nt region drives robust amplification without requiring perfect sequence matching to the genome. The remaining 5′ portion of Primer A functions as an anchor sequence for Primer B in the targeted amplification step and for Primer E in the adapter primer amplification step (See Figure 1). All PCR steps in this protocol are performed using the maximum ramp rate of the thermal cycler.

See Figure 1, step 1. This step comprises two sequential rounds of linear amplification applied directly to the bisulfite-converted template. By utilizing a dNTP/dUTP mixture, uracil bases are actively incorporated into the amplicons. This uracil labeling enables UDG, after the subsequent targeted PCR, to selectively degrade Primer A, primer dimers, and non-specific products before they enter the final adapter PCR and sequencing. By preventing these by-products from being amplified and consuming sequencing reads, this strategy preserves on-target reads and thereby maximizes the effective recovery of target molecules.

1. Prepare the Linear Amplification mixture in PCR 8-Strip Tubes on ice as follows:

**Table:** 

Reagent	Amount
Bisulfite-converted DNA	21 μL
NEBuffer 2 (10X)	3 μL
dNTP/dUTP Mix	3 μL
Primer A Mix (5 μM)	2 μL
Total	29 μL

2. Vortex the mixture gently (∼600 rpm) for 5 s and centrifuge at 5,000 rpm for 3 s.
3. Incubate the tubes in a thermal cycler at 95 °C for 1 min, followed by a 4 °C hold to allow the semi-random primers to anneal to the template DNA.
4. Centrifuge at 5,000 rpm for 10 s to collect the contents at the bottom of the tube.
5. Carefully open the caps and add 1 μL of Klenow Fragment (exo-) (5 U/μL) to each sample on ice.
6. Mix by gently pipetting up and down and centrifuge at 5,000 rpm for 3 s.

***Note:*** Klenow Fragment (exo-) is used for two key biochemical reasons. First, it has strong strand-displacement activity and remains active at low temperatures, enabling isothermal synthesis directly from semi-random primers annealed to the single-stranded bisulfite-converted template. Second, it lacks 3′→5′ exonuclease activity, which prevents degradation of the semi-random/UMI primer ends and preserves the incorporated dU bases essential for the downstream step.

**CRITICAL:** Do not add the Klenow Fragment (exo-) to the initial reaction mixture in Step 1. The 95 °C denaturation step will irreversibly inactivate the enzyme. It must only be added after the samples have cooled to 4 °C.

7. Perform the First Round Linear Amplification thermal cycling program as follows:

**Table:** 

Temperature	Time
4 °C	50 s
10 °C	1 min
20 °C	4 min
30 °C	4 min
37 °C	4 min
95 °C	30 s
4 °C	hold

***Note:*** The 95 °C step at the very end of this first round denatures the newly synthesized dUTP-containing strand from the genomic template, allowing the template for fresh primers to anneal during the subsequent step.

8. Centrifuge at 5,000 rpm for 10 s to collect the contents at the bottom of the tube.
9. Carefully open the caps. Add the following reagents to the reaction product on ice:

**Table:** 

Reagent	Amount
Reaction Product (from Step 5)	30 μL
NEBuffer 2 (10X)	0.25 μL
dNTP/dUTP Mix	0.1 μL
Nuclease-Free Water	1.15 μL
Klenow Fragment (exo-) (5 U/μL)	1 μL
Total	32.5 μL

**CRITICAL:** The individual volumes of each reagent added in this step are very small, which can lead to pipetting error and inconsistency across samples. To minimize this, prepare a Master Mix of the add-in reagents and add 2.5 μL of this Master Mix to each sample.

10. Vortex the mixture gently for 5 s and centrifuge at 5,000 rpm for 3 s.
11. Perform the Second Round Linear Amplification thermal cycling program as follows:

**Table:** 

Temperature	Time
4 °C	50 s
10 °C	1 min
20 °C	4 min
30 °C	4 min
37 °C	4 min
4 °C	hold

12. Purify the linear amplification products with 4× AMPure XP beads (130 μL).
  a. Allow the AMPure XP beads to equilibrate to 20 °C for at least 30 min before use.
  b. Vortex the beads at 2,000 rpm for 30 s to resuspend.
  c. Add 130 μL to sample and vortex thoroughly.
  d. Centrifuge at 5,000 rpm for 1 s and incubate at 20 °C for 10 min.
  e. Place the samples on a magnetic rack about 5 min until the solution is clear, then carefully remove supernatant.
  f. On magnet, add 200 μL of 80% ethanol (prepared the same day) and pipette up and down 5 times to rinse the beads.
  g. Incubate at 20°C for 30 s, then carefully remove supernatant.
  h. Repeat Step e-f once.
  i. Air-dry the beads with caps open on magnet for 5 min.
  j. Remove from the magnet. Add 20 μL Nuclease-Free Water, vortex thoroughly.
  k. Incubate at 20 °C for 2 min.
  l. Place on the magnet until the solution is clear, then carefully transfer 19 μL of the supernatant to a new tube.

**Pause point:** Store the product at −20 °C until next step.

### Targeted amplification

**Timing: 1.5 h**

See Figure 1, steps 2 and 3. During this step, a multiplexed targeted PCR specifically enriches the selected mCGCGCGG-CpG markers using a dUTP-tolerant DNA polymerase. Immediately post-amplification, UDG enzyme selectively cleaves the uracil residues integrated into the linear amplification primers and off-target templates, leaving the newly synthesized, dimer-free target amplicons completely intact.

13. Prepare the Targeted PCR mixture on ice as follows:

**Table:** 

Reagent	Amount
Purified DNA (from Step 12)	19 μL
Primer B (10 μM)	1 μL
Locus-specific Primer Mix (1 μM each)	5 μL
KAPA HiFi HotStart Uracil+ ReadyMix (2X)	25 μL
Total	50 μL

**CRITICAL:** Quantify each locus-specific target primer (Table S1) after dissolution in TE buffer using a UV spectrophotometer (e.g., Nanodrop), as the concentrations provided by the primer synthesis company are only approximate. Normalize all target primers to the same concentration (1 μM each) before pooling to minimize inter-target variation between primer batches. Prepare a master mix of these reagents and aliquot it into each sample to ensure accurate pipetting.

***Note:*** Primer B acts as a universal forward primer by hybridizing to the common tail sequence introduced by Primer A during the preceding linear amplification step.

14. Vortex gently for 3 s and centrifuge at 5,000 rpm for 3 s. Perform the thermal cycling program as follows:

**Table:** 

Temperature	Time	Cycles
98°C	45 sec	1
98°C	15 sec	10
60°C	30 sec	
72°C	30 sec	
72°C	1 min	1
4°C	forever

15. Centrifuge at 5,000 rpm for 10 s. Add 1 μL UDG enzyme (1 U/μL) to the 50 μL reaction.
16. Vortex gently for 3 s and centrifuge at 5,000 rpm for 3 s.
17. Incubate in a thermal cycler at 37 °C for 30 min, followed by a 4°C hold.

***Note:*** The UDG enzyme degrades Step 1's uracil-containing primers and products but preserves the new targeted amplicons because the KAPA amplification mix utilizes standard dTTP rather than dUTP.

18. Purify the reaction using 153 μL (3× volume) of AMPure XP beads following the protocol described in Step 12.
19. Elute with 35.5 μL of Nuclease-Free Water and transfer the entire 35.5 μL of supernatant to a new tube.

**Pause point:** Store the product at −20 °C until next step.

### CGCGCGG and adapter primer amplification

**Timing: 2 h**

See Figure 1, step 4. This final PCR reaction captures the targeted CGCGCGG sites and incorporates Illumina-compatible sequencing adapters along with sample-specific indices at both ends.

20. Prepare the final PCR mixture on ice as follows:

**Table:** 

Reagent	Amount
Purified DNA (from Step 19)	35.5 μL
10X Ex Taq Buffer	5 μL
dNTP Mixture (2.5 mM each)	5 μL
Primer C (10 μM)	1 μL
Adapter Primer D (i7 index, 10 μM)	1.25 μL
Adapter Primer E (i5 index, 10 μM)	1.25 μL
TaKaRa Ex Taq Hot Start Version	1 μL
Total	50 μL

21. Vortex gently for 5 s and centrifuge at 5,000 rpm for 3 s. Perform the thermal cycling program as follows:

**Table:** 

Temperature	Time	Cycles
95 °C	3 min	1
50 °C	30 sec	
72 °C	1 min	
95 °C	30 sec	20
64 °C	30 sec	
72 °C	1 min	
72 °C	5 min	1
4 °C	forever

***Note:*** Primer C captures the amplicons via its CGCGCGG sequence. The first 3 steps (95 °C for 3 min, 50 °C for 30 sec, and 72 °C for 1 min) allow Primer C to anneal and extend prior to adapter amplification. Primer D anneals to the single-extension product of Primer C, while Primer E anneals to the Primer A anchor sequence, enabling exponential amplification to generate the fully structured illumina sequencing library (See Figure 1, step 4). Twenty cycles of adapter amplification are recommended for an initial input of 6 ng cfDNA; this can be optimized based on the starting mass.

22. Centrifuge at 5,000 rpm for 10 s. Purify the reaction using 60 μL (1.2× volume) of AMPure XP beads following the procedure described in Step 12.
23. Elute with 25 μL of Nuclease-Free Water and transfer the entire 25 μL of the final library to a new tube.
24. Quantify the dsDNA concentration of the final library using the Qubit dsDNA HS Assay Kit.

***Note:*** For an initial input of 6 ng cfDNA followed by 20 amplification cycles, the expected total yield of the final library should be approximately 200 ng.

### Quality control and sequencing

**Timing: 2–7 days**

This step evaluates the concentration and fragment size distribution of the final libraries. Qualified libraries are subsequently normalized and pooled by equal mass to ensure uniform sequencing coverage.

25. Quantify the dsDNA concentration of the final libraries using the Qubit dsDNA HS Assay Kit.
26. Assess the fragment size distribution and library purity by sampling a subset of the libraries using a fragment analyzer (e.g., Agilent 5200 Bioanalyzer).

**Figure 2:**
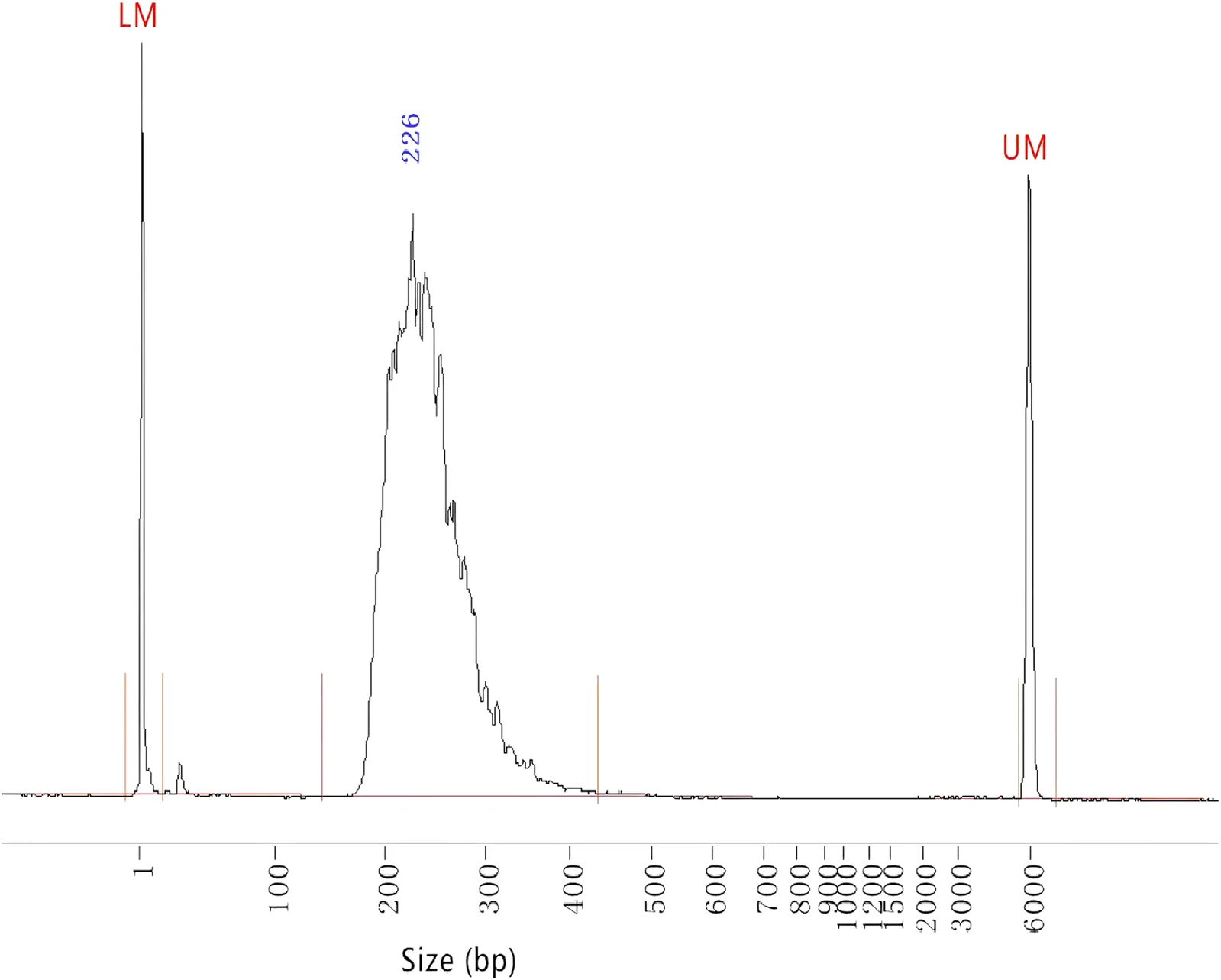
Technical validation of tMCTA-seq A representative electropherogram of the final purified tMCTA-seq library, demonstrating a distinct target amplicon peak with minimal contamination from primer dimers or non-specific products.

***Note:*** A high-quality library displays a distinct amplicon peak corresponding to the targets with minimal peaks in the <150 bp range (Figure 2), confirming effective primer dimer elimination.

27. Construct the multiplexed sequencing pool by combining an equimolar mass of each uniquely indexed library into a single DNA LoBind microcentrifuge tube. Verify that no two libraries share identical i5/i7 index combinations.
28. Sequence the pooled library on an Illumina platform (e.g., NovaSeq X) using a paired-end sequencing strategy (PE150), with 2 Gb of raw data per sample.

### Data processing

**Timing: Variable**

The complete bioinformatics pipeline, including all custom Python and R scripts, is publicly available on GitHub: https://github.com/Guo-Yuqing/tMCTA-seq.

29. Trim sequencing adapters and procedural primers.
  a. Strip terminal Illumina adapter sequences utilizing the Cutadapt software (v3.4), with a maximum error tolerance rate of 10%, which is the default setting according to the user guide of Cutadapt.
    cutadapt -a GATCGGAAGAGCACA -A GATCGGAAGAGCGTC -e 0.1 \
     o out.R1.fastq -p out.R2.fastq \
      untrimmed-output out.R1. untrimmed fastq
      untrimmed-paired-output out.R2. untrimmed fastq
    raw.R1.fastq raw.R2.fastq \
  b. For reads lacking detectable adapters, trim 14 bp from the ends to remove potentially degraded or low-quality adapter remnants.
  c. Remove specific tMCTA-seq procedural primers:
    i. Trim 12 bp from the 5′ end and 6 bp from the 3′ end of Read 1 (R1).
    ii. Trim 6 bp from the 5′ end and 12 bp from the 3′ end of Read 2 (R2).
30. Quality control and bisulfite conversion filtering
  a. Filter low-quality reads using Fastp (v0.23.0).
    fastp -i trimmed.R1.fastq -I trimmed.R2.fastq \-o clean.R1.fastq -O clean.R2.fastq \-A -q 20 -u 50 -n 5 -l 37
    ***Note:*** These parameters mandate the rejection of read pairs if fewer than 50% of bases achieve a Phred quality score ≥ 20 (-u 50 -q 20), if ambiguous 'N' bases exceed a count of 5 (-n 5), or if the read length falls below 37 bp (-l 37). The auto-adapter trimming heuristic (-A) is concurrently engaged.
  b. Enhance the precision by strictly excluding any reads containing > 3 unmethylated CHs (CC, CA, CT) to eliminate incompletely converted genomic templates.
31. Sequence alignment and post-alignment filtering
  a. Map the clean reads to the human reference genome (hg19) using Bismark (v0.16.3).
    bismark --bowtie1 --non_directional --genome/path/to/hg19_index \
      fastq --phred33-quals clean.R1.fastq
    bismark --bowtie1 --non_directional --genome/path/to/hg19_index \
      fastq --phred33-quals clean.R2.fastq
  b. Sort the aligned.bam files by coordinate using Samtools (v1.3.1).
  samtools sort -o sorted.bam aligned.bam
  c. Retain only aligned reads containing ≥ 2 CpG sites within ± 3 bp of the 5′-end to remove non-specific amplifications.
32. Deduplicate PCR amplification artifacts utilizing UMIs.
  a. Extract the 5 bp UMI (HHHHH) from the 5′ end of each R1 read.
  b. Retain reads with quality scores > 20 for all 5 UMI bases; discard others.
  c. Group reads by UMI sequence and alignment coordinates, correcting for 1-bp mismatches in UMI sequences.
  d. Remove PCR duplicates within each group, retaining one representative read per deduplicated UMI group.
  e. Discard any UMI group supported by fewer than 2 raw reads.
33. Compute absolute methylation values.

Summarize target CpG regions by calculating the total UMIs. Generate diagnostic datasets using custom R scripts. For examples of methylation values for plasma samples, see Table S2 in Jiang et al.^10^

34. Prediction scores for CRC and GC.

Ensemble models for CRC and GC detection were constructed using predictions generated from a 5-fold, out-of-fold (OOF) procedure on the training set of each cancer cohort. Given the constructed sample-by-marker feature matrix, follow the released prediction notebooks in the tMCTA-seq analysis pipeline (‘crc_model_prediction.ipynb’ for CRC and ‘gc_model_prediction.ipynb’ for GC) to load the pre-trained ensemble models and calculate a prediction score for each sample. Samples with a CRC prediction score ≥ 0.34 are classified as CRC-positive, and samples with a GC prediction score ≥ 0.38 are classified as GC-positive.

35. Differentiation between CRC and GC in blood.

For patients who were positive for both CRC and GC prediction scores, calculate the ratio of the total UMI counts of the CRC-specific markers (CRC versus GC, CvsG, n = 22) to those of the GC-specific markers (GC versus CRC, GvsC, n = 24). Using a log-ratio cutoff of 0, classify the patient as CRC or GC.

## Expected outcomes

A successful execution of the tMCTA-seq protocol will yield high-quality targeted library free of primer dimers, owing to the dUTP-UDG strategy. Automated electrophoresis should display a distinct amplicon peak with minimal low-molecular-weight contamination (Figure 2A). The assay achieved an average molecule capture efficiency of approximately 23% and exhibited a robust limit of detection down to 2.3 pg (<1 haploid genome).^10^

**Figure 3:**
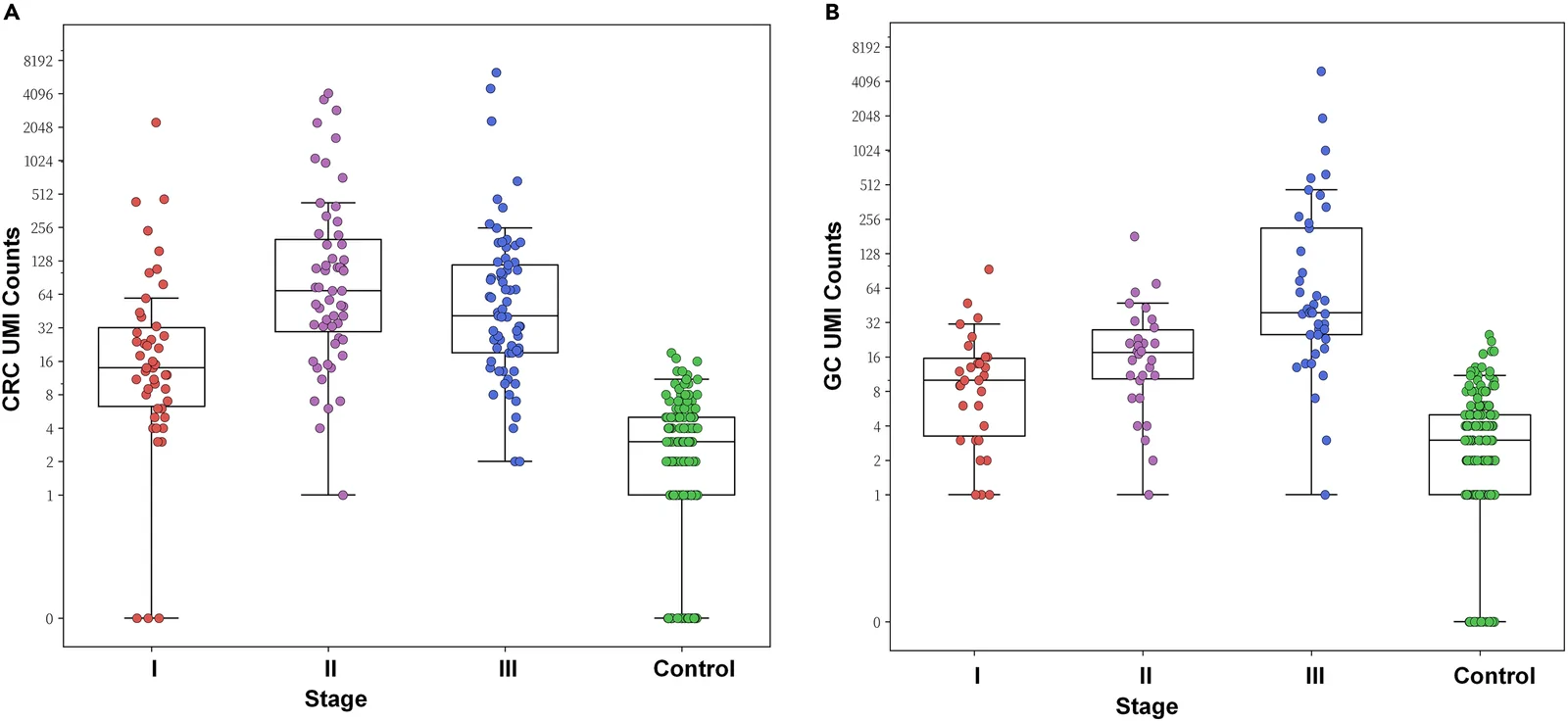
Total UMI counts for CRC and GC (A) The total UMI counts of the 72 mCGCGCGG-CpG markers in CRC patients and control participants. (B) The total UMI counts of the 68 markers in GC patients and control participants. The X-axis labels indicate the clinical stage for cancer patients; “Control” denotes healthy individuals. Stages follow the AJCC 8th edition staging system.

Upon bioinformatics analysis, the diagnostic efficacy for CRC and GC can be directly evaluated using a straightforward “Total UMI Count Method”, defined as the total UMI sum of cancer-specific markers (72 for CRC and 68 for GC). Users should expect the total UMI counts in plasma samples from both early- and advanced-stage cancer patients to be significantly elevated compared to healthy controls. Specifically, the total UMI counts for CRC patients are typically much higher than those for controls (e.g., median UMI counts of 14, 69, and 41 for stages I, II, and III CRC patients, respectively, compared to 3 for the controls) (Figure 3A). For GC, the total UMI counts across all patient stages are also significantly higher (e.g., median UMI counts of 10, 17.5, and 39 for stages I, II, and III GC patients, respectively, compared to 3 for the controls) (Figure 3B).

**Figure 4:**
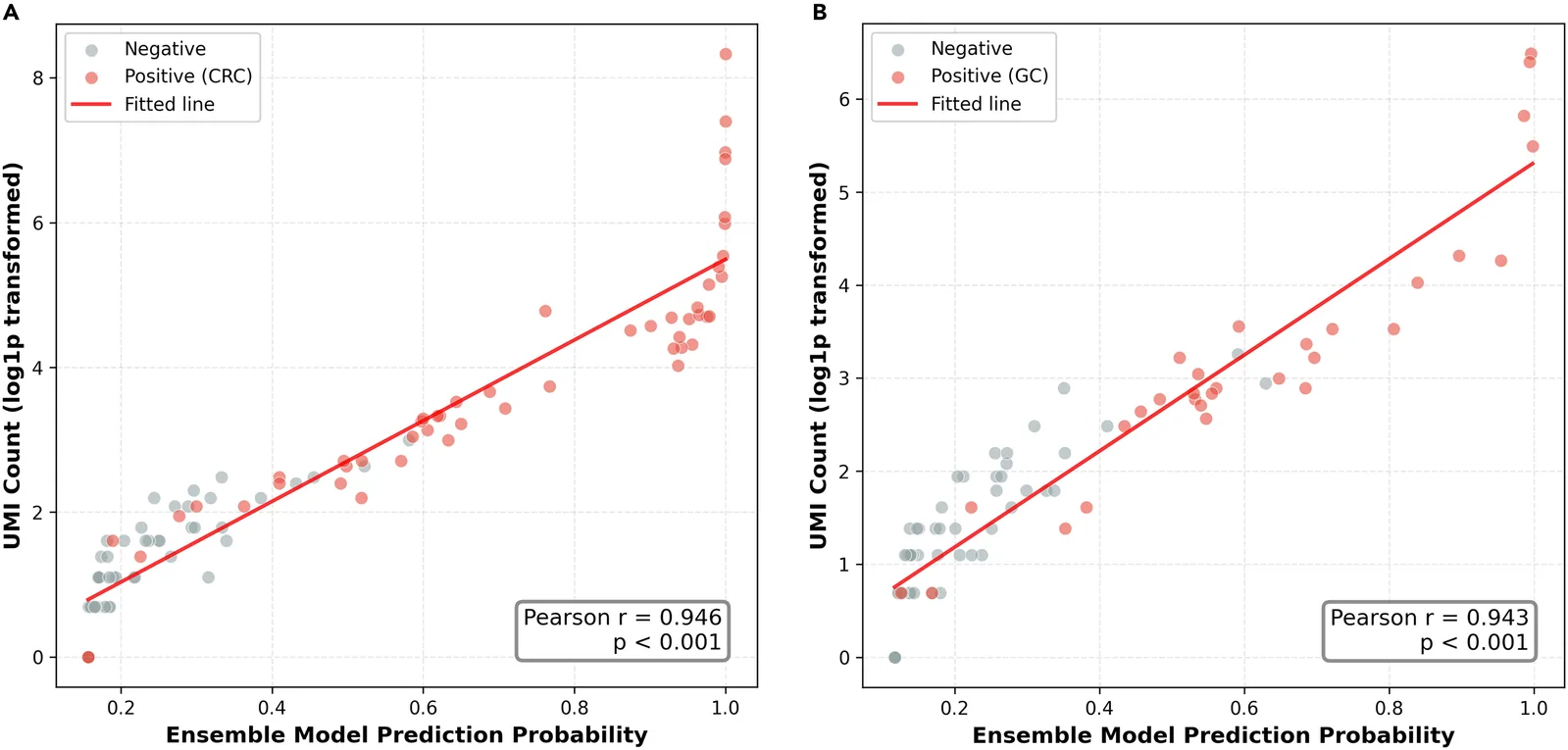
Correlation between the ensemble model prediction scores and UMI values (A) Scatter plot showing the correlation between the ensemble model prediction scores and the total UMI counts of the 72 markers for CRC in the test set. (B) Scatter plot showing the correlation between the ensemble model prediction scores and the total UMI counts of the 68 markers for GC in the test set.

We have established ensemble machine-learning models for CRC and GC detection using predictions generated from a 5-fold, out-of-fold (OOF) procedure on the training set of each cancer patient cohort. In the test set, the models yielded an overall AUC of 0.928 for CRC (88.2% sensitivity, 90.7% specificity) and 0.926 for GC (86.7% sensitivity, 94.4% specificity). Although the ensemble models improve diagnostic performance relative to the “Total UMI Count Method”, their prediction scores show a strong correlation with total UMI counts in both CRC and GC, which confirms their reliability (Figure 4).

Ultimately, tMCTA-seq outperforms routine serological biomarkers (CEA, CA19-9, CA125, and CA72-4).^10^ For CRC, CEA and the four-marker panel yielded sensitivities of only 31.3% and 47.1%, respectively. In contrast, tMCTA-seq detected 88.6% of CEA-negative CRC patients, matching its 87.5% detection rate in CEA-positive cases. Similarly, for GC, tMCTA-seq identified 86.4% of CEA-negative patients, far exceeding the 26.7% and 33.3% sensitivities of CEA and the four-marker panel. Integrating tMCTA-seq with CEA testing boosted sensitivities to >90% for both cancers.

## Limitations

One limitation of this assay relates to its capacity to differentiate between tumor origins. Specifically, the model’s prediction accuracy for distinguishing CRC from GC is slightly lower for CRC than for GC. To mitigate this, we propose a “CRC-prioritized” approach for clinical implementation. Under this differentiation strategy, all individuals who test positive for CRC markers—regardless of their subsequent GC classification score—are recommended for primary colonoscopy. Given that blood-based CRC screening is a well-established and clinically approved practice, this fallback strategy maximizes the diagnostic safety and sensitivity for CRC.

A second limitation is that the current tMCTA-seq assay targets only two cancer types, CRC and GC. By contrast, many blood-based multi-cancer detection methods are designed to detect a broader range of malignancies, including CRC, GC, hepatocellular carcinoma, pancreatic cancer, and esophageal cancer, among others.^4^^,^^11^ Multi-cancer screening generally requires a high-specificity strategy to minimize overdiagnosis. However, such a strategy may not be optimal for blood-based detection of CRC and GC. Because positive screening results for these cancers can be readily followed by confirmatory endoscopy, a high-sensitivity strategy is more appropriate to reduce missed diagnoses. Therefore, an assay focused on CRC and GC has distinct clinical value. In addition, tMCTA-seq could be further expanded by incorporating previously identified mCGCGCGG-CpG markers for other cancer types.^7^^,^^12^

## Troubleshooting

### Problem 1

Atypical fragment size distribution, inadequate cfDNA yield, or low final library yield.

Initial extraction yields inadequate amounts of cfDNA from plasma, the final library yield is unexpectedly low, or the capillary electrophoresis profile displays broad smearing rather than the expected sharp 160–170 bp mono-nucleosome peak. Related to cfDNA extraction, steps 1-2; steps 25–26.

### Potential solution

To address low yields or atypical profiles, first ensure blood samples are centrifuged promptly to remove cellular debris. If processing is delayed, use cfDNA stabilization tubes. During extraction, ensure full Proteinase K digestion and incubate the elution buffer on the silica membrane for 2–3 min to maximize short fragment recovery. If final library yields are low despite successful extraction, verify that the cfDNA input before bisulfite conversion is at least 1 ng. To prevent non-specific DNA loss, add carrier RNA during the bisulfite conversion step. Furthermore, confirm that two rounds of linear amplification were completed and that the KAPA HiFi HotStart Uracil+ enzyme was used during targeted amplification, as standard polymerases will fail to amplify uracil-containing templates. Finally, ensure strict adherence to all detailed steps in the protocol.

### Problem 2

Positive result observed across the entire sample batch.

The entire batch of clinical samples shows suspiciously positive UMI signals during downstream analysis. Related to Control setup, step 7.

### Potential solution

First, ensure that every experimental batch includes both a positive control (FMG spiked into fragmented, cfDNA-sized WBC gDNA) and a negative control (fragmented WBC gDNA only). Check the negative control first; if it shows a positive signal, systemic contamination has occurred. This issue typically arises from amplicon aerosol contamination or improper handling of the FMG control. To prevent this, strict spatial segregation is essential: Targeted Amplification (starting from Step 12) and all subsequent procedures must be conducted in a completely separate room from the pre-amplification steps. Additionally, handle the high-concentration FMG positive control with extreme caution, as it easily causes cross-contamination. Finally, strictly adhere to molecular diagnostic standards by thoroughly decontaminating workstations, pipettes, and equipment with DNA-degrading solutions and UV irradiation immediately after each experiment.

### Problem 3

Electropherogram dominated by low-molecular-weight primer dimers.

The Agilent Bioanalyzer trace displays significant low-molecular-weight peaks (<100 bp) or unexpected broad smears, indicating severe contamination from primer dimers or non-specific products in the final library. Related to step 1, 15–17.

### Potential solution

Ensure the dNTP/dUTP mix is formulated correctly. Confirm the UDG was correctly added in Step 12 and that the enzyme batch is fully active. Verify that the thermal cycler reached and maintained 37 °C for the full 30 min to allow UDG to degrade the dUTP-incorporated non-specific templates. Prepare fresh primer aliquots, as repeated freeze-thaw cycles degrade primers and promote non-specific binding.

### Problem 4

Insufficient UMI recovery despite successful library construction.

The library yield and fragment size appear normal, but the overall number of captured UMIs in the samples is unexpectedly low. Related to step 4–6, 9.

### Potential solution

First, check if the positive control also shows low UMI counts. If so, primer degradation from repeated freeze-thaw cycles is likely. To prevent this, use HPLC-purified primers, aliquot them upon receipt, and use each aliquot only once. Next, ensure the Klenow Fragment is added only after the sample completely cools. Adding it at 95 °C will irreversibly inactivate the enzyme. Finally, during bead purification, ensure precise pipetting and avoid over-drying the beads (until they crack), as this severely decreases the elution efficiency.

## Resource availability

### Lead contact

Further information and requests for resources and reagents should be directed to and will be fulfilled by the lead contact, Lu Wen (wenlu@pku.edu.cn).

### Technical contact

Technical questions on executing this protocol should be directed to and will be answered by the technical contact, Lu Wen (wenlu@pku.edu.cn).

### Materials availability

This study did not generate new materials.

### Data and code availability

This study did not generate new datasets or code.

## Acknowledgments

This work is supported by the 10.13039/501100007129Shandong Provincial Natural Science Foundation (no. ZR2026QC1764), the National High Level Hospital Clinical Research Funding, the Elite Medical Professionals Project of 10.13039/501100012173China-Japan Friendship Hospital (no. ZRJY2023-QM13), and the 10.13039/501100004826Beijing Natural Science Foundation (no. 7244406).

## Author contributions

Z.J. and Y.L. developed the methodology, conducted the investigation, and validated the results. Y.G., K.Q., and X.L. developed the software, curated the data, and performed formal analysis. J.R. contributed to methodology development. R.L. and W.F. provided essential resources. L.W. and X.Z. conceptualized the study and supervised the research. Z.J., Y.G., Y.L., K.Q., X.L., R.L., and J.R. drafted the original manuscript. L.W. and X.Z. reviewed and edited the manuscript. All authors read and approved the final protocol.

## Declaration of interests

The authors declare no competing interests.

## Supplemental information

Table S1. 110 mCGCGCGG-CpG marker characteristics, related to Step 13
